# L265P Mutation of the *MYD88* Gene Is Frequent in Waldenström’s Macroglobulinemia and Its Absence in Myeloma

**DOI:** 10.1371/journal.pone.0080088

**Published:** 2013-11-05

**Authors:** Naoki Mori, Mari Ohwashi, Kentaro Yoshinaga, Kenjiro Mitsuhashi, Norina Tanaka, Masanao Teramura, Michiko Okada, Masayuki Shiseki, Junji Tanaka, Toshiko Motoji

**Affiliations:** Department of Hematology, Tokyo Women’s Medical University, Tokyo, Japan; IRCCS National Cancer Institute, Italy

## Abstract

L265P mutation in the *MYD88* gene has recently been reported in Waldenström’s macroglobulinemia; however the incidence has been different according to the methods used. To determine the relevance and compare the incidence by different methods, we analyzed the L265P mutation in bone marrow mononuclear cells from lymphoid neoplasms. We first performed cloning and sequencing in 10 patients: 8 Waldenström’s macroglobulinemia; 1 non-IgM-secreting lymphoplasmacytic lymphoma; and 1 low grade B-cell lymphoma with monoclonal IgG protein. The L265P mutation was detected in only 1/8 Waldenström’s macroglobulinemia patients (2 of 9 clones). To confirm these results, direct sequencing was performed in the 10 patients and an additional 17 Waldenström’s macroglobulinemia patients and 1 lymphoplasmacytic lymphoma patient. Nine of 28 patients (7/25 Waldenström’s macroglobulinemia, 1/2 lymphoplasmacytic lymphoma, and B-cell lymphoma) harbored the mutation. We next tested for the mutation with *BSi*E1 digestion and allele-specific polymerase chain reaction in the 28 patients and 38 patients with myeloma. Aberrant bands corresponding to the mutation were detected by *BSi*E1 digestion in 19/25 patients with Waldenström’s macroglobulinemia (76%), 1/2 lymphoplasmacytic lymphoma and B-cell lymphoma, but not in the 38 myeloma patients. The L265P mutation was more frequent in patients with Waldenström’s macroglobulinemia than in those with myeloma (p=1.3x10^-10^). The mutation was detected by allele-specific polymerase chain reaction in 18/25 Waldenström’s macroglobulinemia patients (72%). In the 25 Waldenström’s macroglobulinemia patients, the L265P was more frequently detected by *BSi*E1 digestion than by direct sequencing (p=5.3x10^-4^), and in males (15/16, 94%) than in females (4/9, 44%) (p=1.2x10^-2^). No siginificant difference was observed in the incidence of the L265P mutation between *BSi*E1 digestion and allele-specific polymerase chain reaction (p=0.32). These results suggest that the L265P mutation is involved in the majority of Waldenström’s macroglobulinemia. *BSi*E1 digestion and allele-specific polymerase chain reaction may detect a small fraction of mutated cells in some cases.

## Introduction

Waldenström’s macroglobulinemia (WM) is an IgM-secreting-lymphoproliferative neoplasm [1-3]. Monoclonal IgM protein (IgM M-protein) is observed in WM, diffuse large B-cell lymphoma (DLBCL), chronic lymphocytic leukemia, monoclonal gammopathy of undetermined significance (MGUS), and autoimmune disease. Pathologically, patients with WM have lymphoplasmacytic lymphoma (LPL); therefore, they can be distinguished from other lymphoma patients with IgM M-protein [2]. Patients with IgM MGUS progress to WM or other B-cell lymphoma. Until recently, molecular findings associated with the disease were not well characterized in WM. 

Myeloid differentiation primary response gene 88 (MYD88) is an adaptor protein that mediates toll and interleukin (IL)-1 receptor signaling [4-6]. After the toll-like receptor or IL-1 receptor binds to its ligand, Toll/IL-1 receptor (TIR) domains trigger MYD88 through TIR domain containing adaptor protein and Bruton’s tyrosine kinase or activate MYD88 directly. Subsequently MYD88 dimerizes and induces the phosphorylation of IL-1 receptor-associated kinase (IRAK)4, IRAK1, and IRAK2, leading to inhibitor κBα phosphorylation and the activation of nuclear factor (NF)-κB [7,8]. A recent study using RNA interference screening revealed that MYD88, IRAK1, and IRAK4 were essential for survival of activated B-cell-like subtype of DLBCL cells [9]. Sequencing uncovered that the *MYD88* gene was mutated in activated B-cell-like DLBCL tumors [9]. The most frequently found mutation of the *MYD88* gene was a T to C transition at nucleotide 978 (T978C mutation, 29%) resulting in a leucine to proline substitution at amino acid position 265 (L265P). This mutation was located in the TIR domain and was rare in other DLBCL subtypes. The L265P mutation triggers IRAK-mediated NF-κB signaling. Other mutations were also found in the TIR domain in the study, but at a lower frequency. Moreover, *MYD88* gene mutations were found by whole-exome sequencing in 6 of 55 DLBCL patients (11%) [10]. Somatic mutations of the *MYD88* gene were also found in 6 of 46 patients with splenic marginal zone lymphoma (13%) [11]. Non-synonymous *MYD88* mutations were observed in 3 of 53 mucosa-associated lymphoid tissue lymphoma patients (6%) [12].

NF-κB signaling is important for the growth and survival of WM cells [13]. The L265P mutation was recently identified in 49 of 54 patients with WM (91%), and 3 of 3 patients with non-IgM-secreting LPL [14]. Inhibition of MYD88 signaling reduced inhibitor κBα and NF-κB p65 phosphorylation, and NF-κB nuclear staining in WM cells expressing MYD88 L265P [14]. Subsequently the mutation was reported to be present in 18 of 27 patients with WM (67%), and was less frequent in marginal zone lymphoma patients [15]. Most recently, the high incidence of the L265P mutation (93-100%) was reported using the sensitive allele-specific polymerase chain reaction (AS-PCR) in WM [16,17]. These findings suggest that the mutation is useful for distinguishing WM from other diseases or conditions with IgM-M protein. However, whether all patients with WM have the mutation, and which methods are suitable for detecting this mutation have yet to be elucidated in detail. To determine the relevance of the L265P mutation and its association with the clinical characteristics of lymphoid neoplasms, we performed sequencing and mutation analysis on the *MYD88* gene in WM and B-cell lymphoma patients. The mutation was found in the majority of WM patients, and *BSi*E1 digestion and AS-PCR were more sensitive than direct sequencing.

## Materials and Methods

### Patients’ materials

A total of 66 patients with lymphoid neoplasms were analyzed in this study, and consisted of 25 WM, 2 non-IgM-secreting LPL, and 1 low grade B-cell lymphoma with IgG M-protein as well as 38 myeloma (Table 1). Diagnostic criteria by the Second International Workshop on Waldenstrom’s Macroglobulinemia were used in this study [1]. All but one patient had more than 10% bone marrow lymphoplasmacytic infiltration (Table 2). No family history of WM was documented in the patients. Patients with WM1, WM3, WM6, WM7, WM16, and WM25 were administered prednisolone and/or an alkylating agent before bone marrow sampling (Table 2). Mononuclear cells were separated from the bone marrow of hematological neoplasms by Ficoll-Conray gradient centrifugation after obtaining written informed consent. Genomic DNA was prepared by proteinase K digestion and phenol/chloroform extraction or using a QIAamp DNA Blood Mini Kit (Qiagen, Valencia, CA, USA). The current study was conducted within the guidelines and with the approval of the Tokyo Women’s Medical University Ethical Committee, and in accordance with the *Helsinki Declaration*.

**Table 1:** L265P mutation of the *MYD88* gene in lymphoid neoplasms.

Disease	Number of samples	Number of L265P mutation
Waldenström’s macroglobulinemia	25	19
non-IgM-secreting lymphoplasmacytic lymphoma	2	1
low grade B-cell lymphoma with IgG M-protein	1	1
	28	21
myeloma	38	0
Total	66	21

The mutation was detected by *BSi*E1 digestion.

**Table 2:** Clinical characteristics of Waldenström’s macroglobulinemia and lymphoma.

Case	Age/sex	Disease	Serum IgM (mg/dl)	Light chain	Lymphocyte in BM (%)	Karyotype	DS	*BSi*E1 digestion	AS-PCR	Genotype	Allele counts†
WM1	71/Male	WM	1430	κ	29.6	46,XY	-	M	M	MW	1
WM2	64/Female	WM	2302	λ	19.0	NA	-	-	-	WW	0
WM3	41/Female	WM	2218	λ	24.6	46,XX	-	-	-	WW	0
WM4	51/Male	WM	2510	κ	21.0	46,XY	-	M	M	MW	1
WM5	56/Male	WM	1996	κ	47.4	46,XY	M	M	M	MW	1
WM6	74/Female	WM	1910	κ	NA	abnormal1*	-	-	-	WW	0
WM7	65/Male	WM	1095	λ	47.4	46,XY	M	M	M	MW	1
WM8	82/Male	WM	2485	κ	85.6	46,XY	-	M	M	MW	1
WM9	61/Female	WM	2655	κ	43.6	abnormal2*	M	M	M	MW	1
WM10	68/Male	WM	2050	κ	19.8	46,XY	-	M	M	MW	1
WM11	69/Male	WM	1105	κ	44.5	46,XY	M	M	M	MW	1
WM12	52/Female	WM	1264	λ	14.4	NA	-	-	-	WW	0
WM13	72/Male	WM	2840	κ	15.6	46,XY	-	M	M	MW	1
WM14	45/Male	WM	1915	λ	14.1	46,XY	-	M	M	MW	1
WM15	74/Male	WM	2578	κ	96.4	45,X,-Y	M	M	M	MW	1
WM16	78/Female	WM	1142	κ	13.5	46,XX	-	M	M	MW	1
WM17	60/Male	WM	4920	κ	49.0	46,XY	M	M	M	MW	1
WM18	50/Female	WM	1728	λ	25.0	46,XX	-	M	M	MW	1
WM19	76/Male	WM	2234	κ	34.6	46,XY	-	M	-	MW	1
WM20	83/Male	WM	402	κ	24.3	46,XY	-	-	-	WW	0
WM21	63/Male	WM	6942	κ	91.9	46,XY	M	M	M	MW or MM	1 or 2
WM22	77/Male	WM	2008	λ	16.8	45,X,-Y	-	M	M	MW	1
WM23	60/Male	WM	2788	κ	43.6	46,XY	-	M	M	MW	1
WM24	70/Female	WM	2065	κ	89.5	47,XX,+12	-	-	-	WW	0
WM25	72/Female	WM	2471	κ	17.1	46,XX	-	M	M	MW	1
NHL1	45/Male	LPL	7	(κ)	66.7	abnormal3*	-	-	-	WW	0
NHL2	58/Male	LPL	12	(κ)	17.6	46,XY	M	M	M	MW	1
NHL3	64/Female	NHL	31	λ	92.2	NA	M	M	M	MW	1

WM, Waldenström’s macroglobulinemia ; LPL, lymphoplasmacytic lymphoma; NHL, non-Hodgkin’s lymphoma; BM, bone marrow; DS, direct sequencing; AS-PCR, allele-specific polymerase chain reaction; NA, not available; M, L265P mutation; W, wild-type. † L265P allele counts. * Abnormal1, 46,XX,t(11;18)(q21;q21.1)[21]/47,XX,t(11;18)(q21;q21.1),+ del(22q) [2]/46,XX [2]; abnormal2, 46,XX,der(7)t(1;7)(q21;q22)[18]/45,X, - X,del(6)(q11),del(17)(p11.2)[1]/46,XX[1]; abnormal3, 46,XY,del(6)(q22q25-26),t(8;22)(p23;q1?2),der(14)t(11;14)(q13;q32)[3]/ 47,XY,+ 1,der(1;16) (q10;p10),del(6)(q22q25-26),t(8;22)(p23;q1?2),der(14)t(11;14)(q13;q32)[4]/46,XY[27]. NHL1 and NHL2 lack M-protein, and NHL3 is a low grade B-cell lymphoma with IgG M-protein.

### PCR

Sequences of primers for PCR were published previously: 

MY-F, 5'-GGGATATGCTGAACTAAGTTGCCAC3'; and 

MY-R, 5'-GACGTGTCTGTGAAGTTGGCATCTC-3' [14]. 

PCR was performed with 10 ng of DNA. After 5 min at 94 °C, 30 cycles of amplification using 60 s at 94 °C, 60 s at 60 °C, and 60 s at 72 °C were performed, with a subsequent 5 min extension at 72 °C. Primers MY-F and MY-R amplified 726 base pairs (bp) products which cover the TIR domain.

### Sequencing

PCR products were purified using the QIAQuick PCR Purification Kit (Qiagen) and ligated into the pGEM-T vector (Promega, Madison, WI, USA). After cloning, sequencing was performed in both directions on a MegaBase sequence system (Amersham, Buckingham, UK) [18]. Direct sequencing was also performed using purified PCR products.

### *BSi*E1 restriction enzyme digestion

We tested for the L265P mutation with *BSi*E1 restriction enzyme digestion (New England Biolabs, Tokyo, Japan) [15]. The 726 bp PCR products were purified, digested with *BSi*E1, and subjected to electrophoresis through a 2% agarose gel. The presence of both 448 bp and 278 bp products indicated the L265P mutation, while the 726 bp products indicated the wild-type. In each sample, electrophoresis was repeated three to five times using independent PCR products.

### AS-PCR

AS-PCR was performed with specific forward primers with a single base substitution at the end of the primer: MYW-F, 5’-GTGCCCATCAGAAGCGCCT-3’ (wild type) and MYM-F, 5’-GTGCCCATCAGAAGCGCCC-3’ (mutant). Allele-specific primers contained an intentional mismatch at the third nucleotide from the 3’ end to improve specificity [17]. The sequence of the reverse primer was the same as that used for sequencing. PCR was performed with 10 ng of DNA. After 5 min at 94 °C, 35 cycles of amplification using 60 s at 94 °C, 60 s at 65 °C, and 60 s at 72 °C were performed, with a subsequent 5 min extension at 72 °C. Primers MYW-F (or MYM-F) and MY-R amplified 224 bp products. 

### Statistical analysis

Correlations between the frequency of the L265P mutation and type of disease or clinical characteristics were analyzed using the chi-square test or Fisher’s exact probability test. Statistical analysis for the mutation between direct sequencing or AS-PCR and *BSi*E1 digestion was performed with the Wilcoxon’s signed-ranks test. Correlations between the percentage of lymphocytes and presence of L265P or sex were analyzed using the Mann-Whitney’s *U*- test. Correlations between the IgM concentration and presence of L265P were analyzed using the Mann-Whitney’s *U*- test. Statistical analyses were performed using Dr SPSSII (version 11.01) or Statcel 3 software. A P-value of less than 0.05 was considered significant.

## Results

### Sequencing

**Figure 1:**
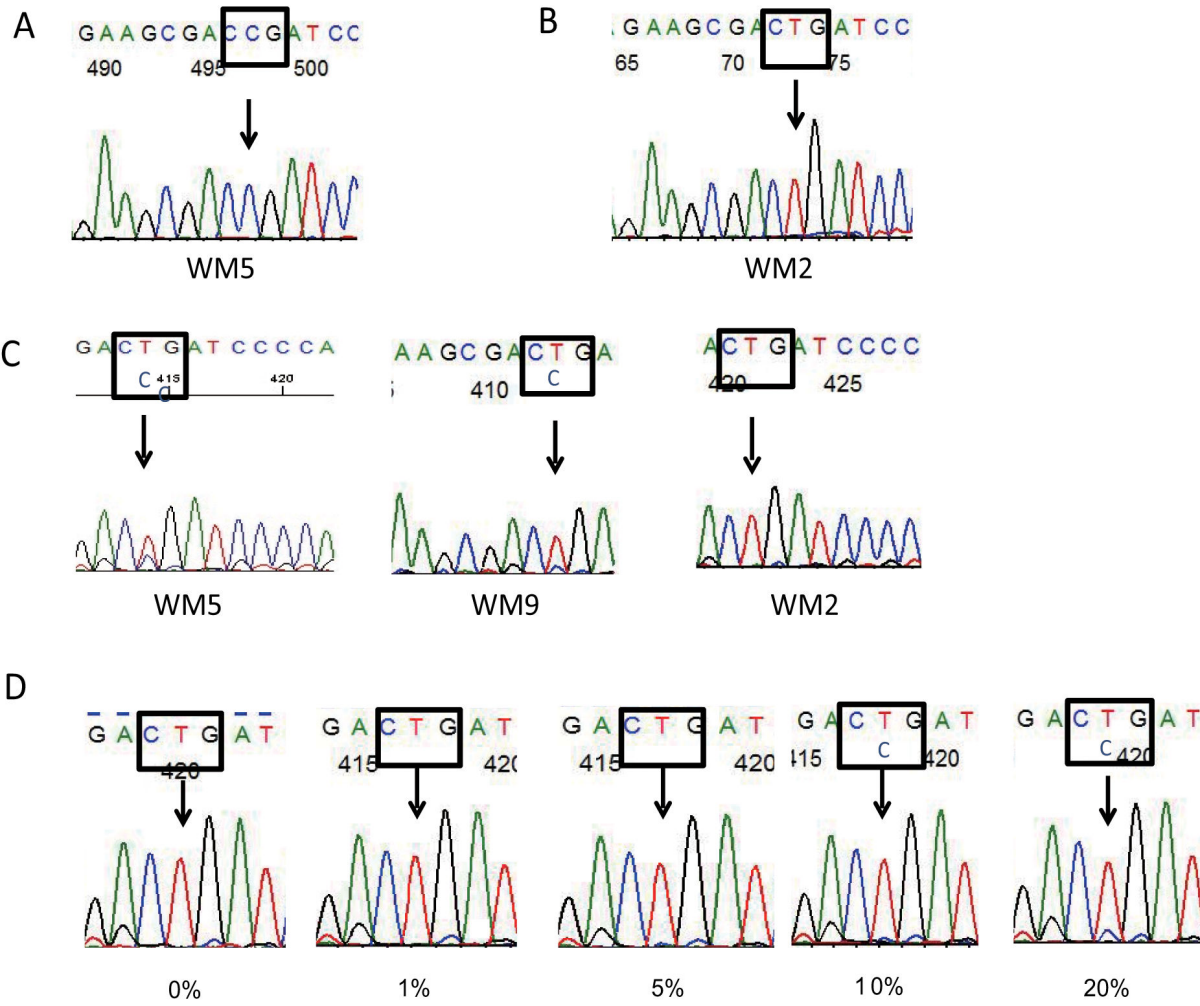
Sequence analysis of the *MYD88* gene in Waldenström’s macroglobulinemia. (A) Sequencing revealed a T to C transition resulting in a leucine to proline substitution at amino acid position 265 (WM5). (B) Wild-type sequences (T) are shown as a control (WM2). (C) Direct sequencing showed both wild-type and mutant alleles in WM5 and WM9, and the wild-type allele only in WM2. (D) Sensitivity of direct sequencing. L265P-positive DNA (WM5) was diluted into wild-type DNA (WM3) before amplification. Aberrant bands were detected in samples containing 10% or more of the L265P mutation.

We first selected 10 patients including 8 WM (WM1-WM8), 1 non-IgM-secreting LPL (NHL1), and 1 low grade B-cell lymphoma with IgG M-protein (NHL3) for cloning (Table 2). Sequencing was performed using at least four clones in each patient. The nucleotide change, the T to C transition resulting in the L265P mutation, was detected in 2 of 9 clones from 1 patient with WM (WM5), while it was absent in any of the clones from the other 9 patients (Figure 1 A-B). Because of the low frequency of the mutation, we next performed direct sequencing in the 10 patients and an additional 17 patients with WM and 1 LPL patient. The T to C transition was detected in 9 of 28 patients (32%) (Table 2, Figure 1 C). It was found in 7 of the 25 WM patients (28%), one of the 2 LPL, and 1 low grade B-cell lymphoma with IgG M-protein. All of the 9 patients with the transition also had wild-type sequences. To determine sensitivity, DNA from the L265P-positive clone (WM5) was serially diluted into DNA from a wild-type clone (WM3) to the following percentages: 0%, 0.1%, 0.5%, 1%, 5%, 10%, 20%, and 30%. Sensitivity to the L265P mutation by direct sequencing was 10% (Figure 1 D). 

### *BSi*E1 restriction enzyme digestion

**Figure 2:**
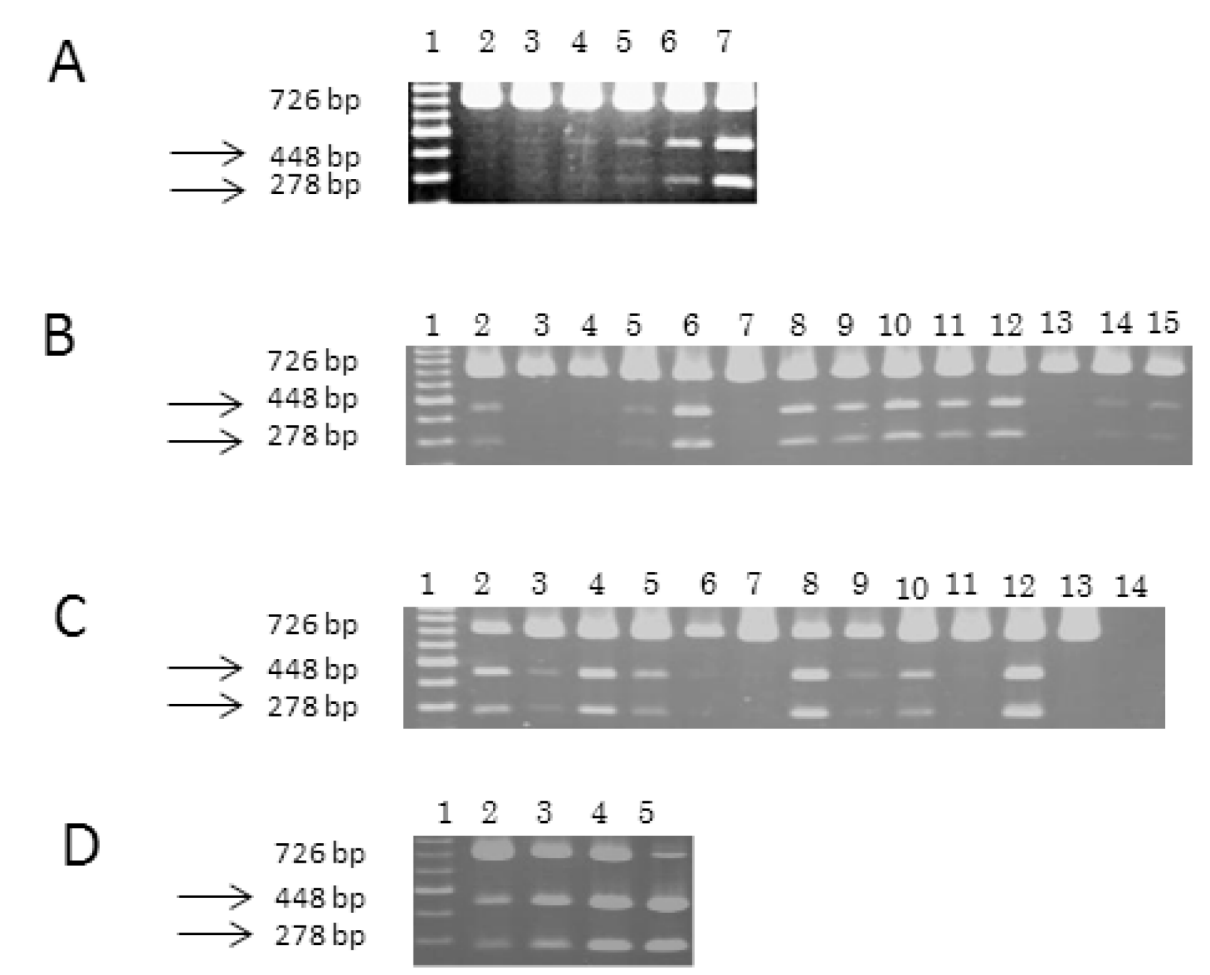
*BSi*E1 digestion of the *MYD88* gene in Waldenström’s macroglobulinemia and lymphoma. Ten μl of PCR products was digested with *BSi*E1, separated by electrophoresis through a 2% agarose gel, stained with ethidium bromide, and visualized by ultraviolet illumination. The size of the products is indicated on the left. (A) Sensitivity of *BSi*E1 digestion. L265P-positive DNA (WM5) was diluted into wild-type DNA (WM3) before amplification. Aberrant bands were detected in samples containing 0.5% or more of the L265P mutation. Lane 1, 100 bp ladder; lane 2, 0%; lane 3, 0.1%; lane 4, 0.5%; lane 5, 1%; lane 6, 5%; lane 7, WM5. (B) Aberrant bands were detected in 10 of 14 samples from WM patients (lanes 2, 5, 6, 8, 9, 10, 11, 12, 14, and 15). Lane 1, 100 bp ladder; lane 2, WM1; lane 3, WM2; lane 4, WM3; lane 5, WM4; lane 6, WM5; lane 7, WM6; lane 8, WM7; lane 9, WM8; lane 10, WM9; lane 11, WM10; lane 12, WM11; lane 13, WM12, lane 14, WM13; lane 15, WM14. (C) Aberrant bands were detected in 8 of 9 samples from WM patients (lanes 2, 3, 4, 5, 6, 8, 9, and 10) and non-Hodgkin’s lymphoma patient (NHL3, lane 12). Lane 1, 100 bp ladder; lane 2, WM15; lane 3, WM16; lane 4, WM17; lane 5, WM18; lane 6, WM19; lane 7, WM20; lane 8, WM21; lane 9, WM22; lane 10, WM23; lane 11, NHL1; lane 12, NHL3; lane 13, normal lymphocyte 1; lane 14, water. (D) Strength of aberrant bands varied in accordance with percentages of L265P-positive DNA. L265P-positive DNA (WM5) was diluted into wild-type DNA (WM3) before amplification. Lane 1, 100 bp ladder; lane 2, 50%; lane 3, 80%; lane 4, 90%; lane 5, 100%.

Since the percentage of lymphocytes (median 27.3%, 13.5-96.4) in the bone marrow varied in each WM patient, the mutation may have been undetectable by direct sequencing in some cases. Thus we tested for the mutation with *BSi*E1 digestion. To determine sensitivity, DNA from the L265P-positive clone (WM5) was serially diluted into DNA from a wild-type clone (WM2 or WM3) to the following percentages: 0%, 0.1%, 0.5%, 1%, and 5%. Sensitivity to the L265P mutation was 0.1-0.5% in our study (Figure 2 A). To compare the sensitivity of direct sequencing with *BSi*E1 digestion, we used the same tube of PCR products for both analyses. We used the same tube of PCR products to screen for the mutation in other samples. The mutation was repeatedly confirmed with different tubes of PCR products. Aberrant bands corresponding to the mutation were detected in 21 of the 28 patients: 19 of the 25 WM (76%), one of the 2 non-IgM-secreting LPL, and B-cell lymphoma with IgG M-protein (Tables 1-2, Figure 2 B-D). All but one patient (WM21) with aberrant bands had hemizygous mutation (Table 2). Of the 21 patients with aberrant bands, 9 had the mutation detected by direct sequencing. Most patients with relatively strong bands were shown to have the mutation by direct sequencing. All of the 9 patients, in whom the L265P mutation was detected by direct sequencing, also showed the mutation by *BSi*E1 digestion (Table 2). To confirm these results, several samples without aberrant bands by digestion were also sequenced; however, only the wild-type sequences were obtained. We also examined in the 38 patients with myeloma with *BSi*E1 digestion; however, no aberrant bands were observed.

### AS-PCR

**Figure 3:**
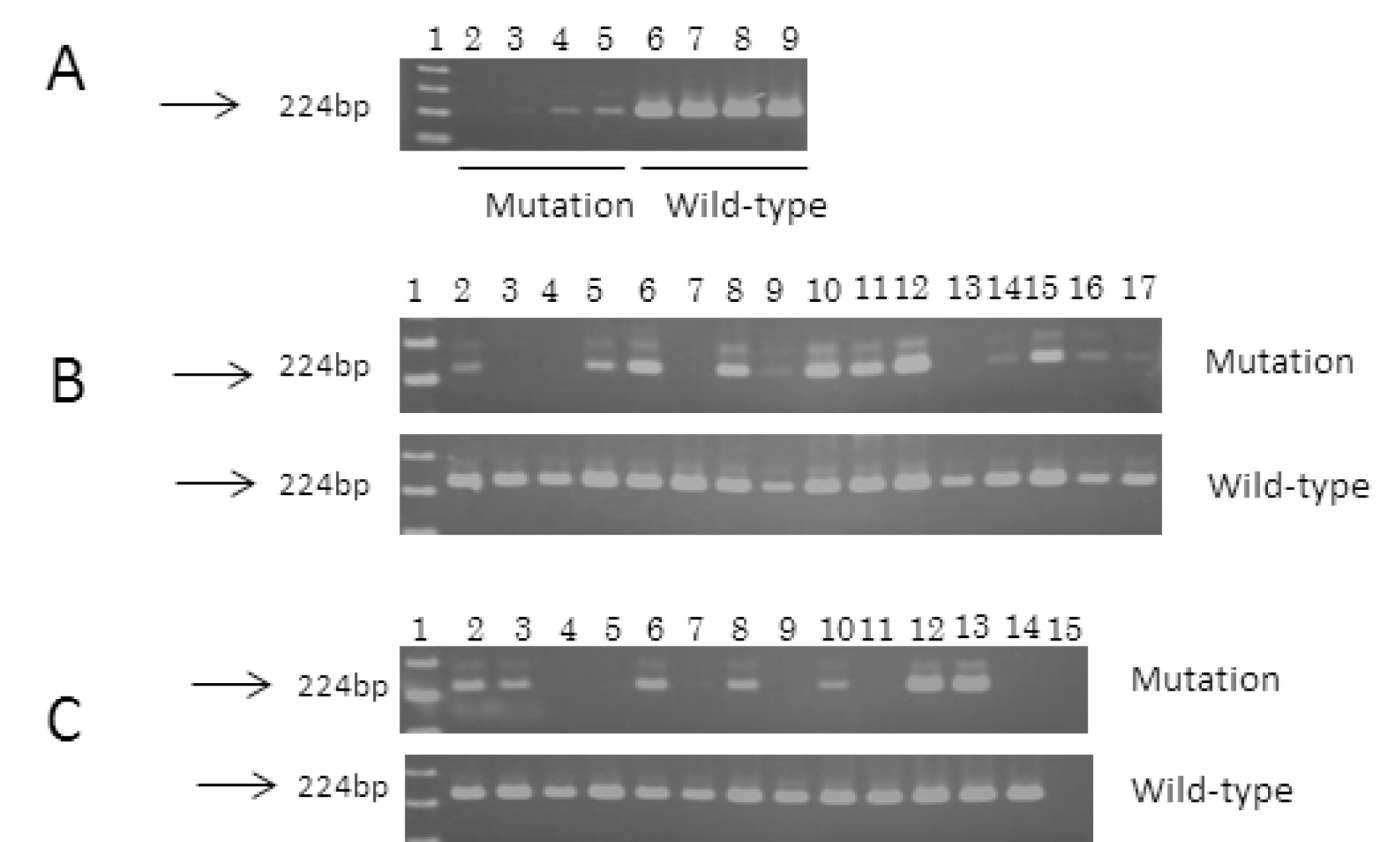
Allele-specific polymerase chain reaction (AS-PCR) of the *MYD88* gene in Waldenström’s macroglobulinemia and lymphoma. Ten μl of PCR products was separated by electrophoresis through a 2% agarose gel, stained with ethidium bromide, and visualized by ultraviolet illumination. The size of the products is indicated on the left. (A) Sensitivity of AS-PCR. L265P-positive DNA (WM5) was diluted into wild-type DNA (WM2) before amplification. Mutations were detected in samples containing 0.1% or more of the L265P mutation. Lane 1, 100 bp ladder; lane 2, 0%; lane 3, 0.1%; lane 4, 0.5%; lane 5, 1%; lane 6, 0%; lane 7, 0.1%; lane 8, 0.5%; lane 9, 1%. (B) Aberrant bands were detected in 12 of 16 samples from WM patients (lanes 2, 5, 6, 8, 9, 10, 11, 12, 14, 15, 16, and 17). Lane 1, 100 bp ladder; lane 2, WM1; lane 3, WM2; lane 4, WM3; lane 5, WM4; lane 6, WM5; lane 7, WM6; lane 8, WM7; lane 9, WM8; lane 10, WM9; lane 11, WM10; lane 12, WM11; lane 13, WM12, lane 14, WM13; lane 15, WM14; lane 16, WM15; lane 17, WM16. (C) Aberrant bands were detected in 6 of 10 samples from WM patients (lanes 2, 3, 6, 7, 8, and 10) and 2 of 3 non-Hodgkin’s lymphoma patients (lanes 12 and 13). Lane 1, 100 bp ladder; lane 2, WM17; lane 3, WM18; lane 4, WM19; lane 5, WM20; lane 6, WM21; lane 7, WM22; lane 8, WM23; lane 9, WM24; lane 10, WM25; lane 11, NHL1; lane 12, NHL2; lane 13, NHL3; lane 14, normal lymphocyte; lane 15, water.

Sensitivity to the L265P mutation by AS-PCR was 0.1-0.5% in our study (Figure 3 A). The mutation was detected in 20 of the 28 patients: 18 of the 25 WM (72%), one of the 2 non-IgM-secreting LPL, and B-cell lymphoma with IgG M-protein (Table 2, Figure 3 B-C). All of the 18 patients, in whom the L265P mutation was detected by AS-PCR, were also shown to have the mutation by *BSi*E1 digestion (Table 2). 

**Figure 4:**
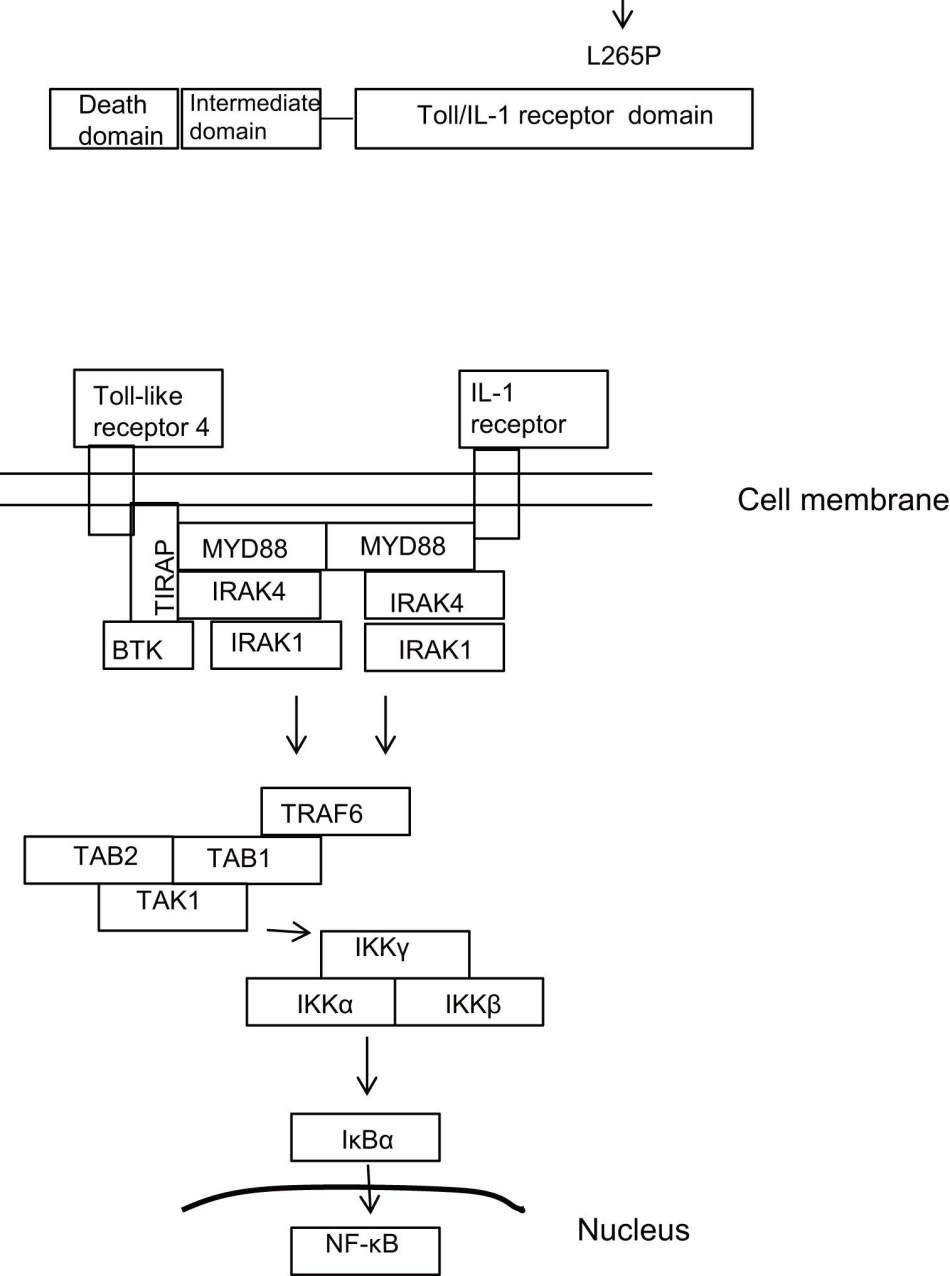
Location of the analyzed mutation and a schema of the involved pathway. TIRAP, TIR domain containing adaptor protein; IRAK, IL-1 receptor-associated kinase; BTK, Bruton’s tyrosine kinase; TRAF, tumor necrosis factor receptor-associated factor; TAK, TGF-β-activated kinase; TAB, TAK binding protein; IκB, inhibitor κB; IKK, IκB kinase; NF-κB, nuclear factor-κB.

The location of the analyzed mutation and a schema of the involved pathway are shown in Figure 4.

### Statistical analysis

#### Analysis between WM and myeloma

The incidence of the mutation by *BSi*E1 digestion was significantly higher in WM than in myeloma (p=1.3x10^-10^, odds ratio=∞) (Table 3).

**Table 3:** Statistical analysis for the L265P mutation.

	Incidence of mutation (%)	P-value	Odds ratio (95% CI)
L265P mutation and type of disease*			
WM versus myeloma	19/25 (76) versus 0/38 (0)	1.3x10^-10^†	∞
L265P mutation in WM*			
Male versus female	15/16 (94) versus 4/9 (44)	1.2x10^-2^†	18.75 (1.68-209.55)
κ chain versus λ chain	15/18 (83) versus 4/7 (57)	0.19	3.75 (0.54-26.19)
	Incidence of mutation (%)	P-value	
L265P mutation and methods in WM			
*BSi*E1 digestion and direct sequencing	19/25 (76) and 7/25 (28)	5.3x10^-4^†	
*BSi*E1 digestion and AS-PCR	19/25 (76) and 18/25 (72)	0.32	
		P-value	
Percentage of lymphocytes in WM			
L265P group and wild-type group*		0.49	
Male and female		0.17	
IgM concentration and L265P mutation in WM			
L265P group and wild-type group*		0.09	

CI, Confidence interval; WM, Waldenström’s macroglobulinemia. * The mutation was detected by *BSi*E1 digestion. †P-value of less than 0.05 was considered significant.

#### Analysis using different genotyping methods in WM

The L265P mutation was more frequently detected in the 25 WM patients, by *BSi*E1 digestion (76%) than by direct sequencing (28%) (p=5.3x10^-4^) (Table 3). No siginificant difference was observed in the incidence of the L265P mutation between *BSi*E1 digestion (76%) and AS-PCR (72%) (p=0.32).

#### Association between clinical characteristics and the L265P mutation by *BSi*E1 digestion in WM

Detection of the L265P mutation by *BSi*E1 digestion was significantly more frequent in males (15/16, 94%) than in females (4/9, 44%) (p=1.2x10^-2^, odds ratio=18.75) (Table 3). The mutation was more frequent in the κ light chain group (15/18, 83%) than in the λ light chain group (4/7, 57%), but this was not significant (p=0.19, odds ratio=3.75). The percentage of lymphocytes was not significantly different between the L265P group and wild-type group (p=0.49). It was not significantly different between male and female patients (p=0.17). The IgM concentration was not significantly different between the L265P group and wild-type group (p=0.09).

## Discussion

The L265P mutation of the *MYD88* gene was detected by direct sequencing in 7 of the 25 patients with WM. It was detectable in 19 of the 25 WM patients by *BSi*E1 enzyme digestion and 18 of the 25 WM by AS-PCR. On the contrary, the L265P mutation was absent in the 38 myeloma patients. A recent study showed that the L265P mutation was found in 91% of WM patients but not in 10 myeloma patients [14]. Our results are consistent with this observation. In the present study, besides the patients with WM, 1 low grade B-cell lymphoma with IgG M-protein had the L265P mutation. Although a previous study showed an association between the mutation and IgM M-protein, our results demonstrated that the L265P mutation was also present in B-cell lymphoma with IgG M-protein [15].

The sensitivity of direct sequencing has been reported to be about 10-20% [19-22]. Sensitivity to the L265P mutation by direct sequencing was 10% in our study. DNA derived from lymphoma cells may be lower than detectable levels in some cases. However, although the mutation was undetectable by direct sequencing in 18 of the 25 WM patients, 9 of the 18 patients had more than 20% lymphocytes before mononuclear cell isolation. In most cases, the L265P mutation in WM was reported to be hemizygous [14]. Therefore direct sequencing can potentially detect the mutation in patients with more than 20% lymphocytes in their bone marrow before mononuclear cell isolation. On the other hand, the sensitivity of *BSi*E1 digestion and AS-PCR was 0.1-0.5% in our study, which indicated that these analyses were more sensitive methods. Although the patient with WM16 was administered prednisolone before sampling and had only 13.5% lymphocytes in her bone marrow, the mutation was detected by *BSi*E1 digestion and AS-PCR. To test the sensitivity of direct sequencing and *BSi*E1 digestion, we used the same tube of PCR products. Although a single clone was used as a sequencing template, Sanger sequencing sometimes showed a small peak in addition to a large peak at several nucleotides. Therefore, it was sometimes difficult to decide whether the wild-types of sequences or additional nucleotide changes were present. In contrast, it was easier to detect aberrant bands corresponding to the mutation using *BSi*E1 digestion, since wild- type DNA did not show aberrant bands.

The incidence of the L265P mutation detected by direct sequencing was lower in the 25 patients with WM than in the recent report [14]. Lymphocyte counts (the percentage of lymphocytes) include lymphoma cells as well as normal lymphocytes. Since the recent study used mononuclear cells sorted by magnetic beads [14], the mutation may have been more frequent than in the present study. In contrast, the proportion of lymphoma cells may have been lower than the detectable levels by direct sequencing in other cases in which the mutation was detected by *BSi*E1 digestion (WM1, WM4, WM8, WM10, WM13, WM14, WM16, WM18, WM19, WM22, WM23, and WM25). Another possibility is that a substantial proportion of lymphoma cells do not harbor the mutation in L265P-positive cases. WM5, which showed aberrant bands by *BSi*E1 digestion, contained 47.4% lymphocytes with lymphoplasmacytic morphology in the bone marrow before mononuclear cell isolation. The L265P mutation was detected by direct sequencing; however, the mutation was only found in two of the 9 clones from WM5. Moreover, although WM8 had aberrant bands by *BSi*E1 digestion and contained 85.6% lymphocytes in the bone marrow, the L265P mutation was not detected by direct sequencing. 

Unexpectedly, the L265P mutation of WM was more frequent in males than in females. A larger percentage of lymphocytes in the bone marrow may have affect the incidence of the mutation; however, it was not significantly different between male and female patients. In addition, although tumor cells with the L265P mutation have growth and survival advantages due to NF-κB activation [13], the percentage of lymphocytes was not significantly different between the L265P group and wild-type group.

The IgM concentration was not significantly different between the L265P group and wild-type group (p=0.09). Although WM is an IgM-secreting-lymphoproliferative neoplasm, no minimal serum IgM level, nor a minimal percentage of bone marrow infiltration is required to establish a diagnosis. Large heterogeneity can exist among patients between their respective serum IgM levels and bone marrow involvement [3]. Moreover, the paraprotein concentration had no prognostic value in most studies, and does not appear to reflect disease bulk in individual patients [2].

The concentration of monoclonal IgM can vary widely in WM patients and it is impossible to define a concentration that reliably distinguishes WM from MGUS [1]. In the recent study, the L265P mutation was found in 2 of 21 patients with IgM MGUS (10%). One of the 2 patients had progressive disease with serial increases in serum IgM and decreases in hematocrit, while the disease was only recently diagnosed in the other patient [14]. This observation suggests that the L265P mutation is associated with the progression from IgM MGUS to WM. Recent studies further support this hypothesis [16,17]. In the present study, some of the patients also had a history of IgM MGUS. Unfortunately, we were unable to collect cells in their MGUS stage to establish the significance of the mutation in the disease progression.

In summary, this study showed that the L265P mutation was frequent in WM patients and absent in myeloma patients. Our results revealed that *BSi*E1 digestion and AS-PCR were more sensitive than direct sequencing. Since some patients only had a small amount of the mutant allele, the contribution of the mutation remains to be clarified in these cases. Further studies need to be conducted to elucidate the genesis and clinical significance of the L265P mutation in WM patients and develop new therapeutic approaches through the NF-κB signaling pathway. 
